# Species identification by experts and non-experts: comparing images from field guides

**DOI:** 10.1038/srep33634

**Published:** 2016-09-20

**Authors:** G. E. Austen, M. Bindemann, R. A. Griffiths, D. L. Roberts

**Affiliations:** 1 Durrell Institute of Conservation and Ecology, School of Anthropology and Conservation, Marlowe Building, University of Kent, Canterbury CT2 7NR, UK; 2 School of Psychology, Keynes College, University of Kent, Canterbury CT2 7NP, UK

## Abstract

Accurate species identification is fundamental when recording ecological data. However, the ability to correctly identify organisms visually is rarely questioned. We investigated how experts and non-experts compared in the identification of bumblebees, a group of insects of considerable conservation concern. Experts and non-experts were asked whether two concurrent bumblebee images depicted the same or two different species. Overall accuracy was below 60% and comparable for experts and non-experts. However, experts were more consistent in their answers when the same images were repeated, and more cautious in committing to a definitive answer. Our findings demonstrate the difficulty of correctly identifying bumblebees using images from field guides. Such error rates need to be accounted for when interpreting species data, whether or not they have been collected by experts. We suggest that investigation of how experts and non-experts make observations should be incorporated into study design, and could be used to improve training in species identification.

Accurate species identification is essential to ecological monitoring^1^,^2^. Species observations are used to inform and evaluate conservation actions^3^, such as the monitoring of population trends^4^,^5^, the implementation and evaluation of population management plans^6^, health assessments of ecosystems^7^, and extinction analysis^8^. Conversely, species *mis*identification can have serious negative impacts, such as the accidental culling of endangered species, exemplified by the threatened takahē *Porphyrio hochstetteri* (Meyer 1883) being mistaken for the destructive pukeko *Porphyrio porphyrio melanotus* (Temminck 1820)^9^, the incorrect monitoring of harmful algal blooms^10^, the unobserved decline in important fish stocks^11^, and wasted resources, such as the drafting of inappropriate management plans from false species sightings^12^.

While species identification in these contexts is conducted routinely by experts, such as taxonomists in museums or academic institutions^13^, there is also a long-standing tradition of members of the public supporting scientific research by contributing identification data^14^. Previously known as amateur naturalists, and more recently as citizen scientists, 70,000 of these lay recorders submit species observations on an annual basis in the UK alone^15^. These observers are recognised as a valuable asset in the monitoring of global environmental change^16^,^17^. However, little is known about the accuracy of species identifications by non-experts, or how these compare to those of experts^18^. Although some doubts have been raised over the ability of volunteers to conduct ‘real research’^19^, the assumption that recorded species have been correctly identified is rarely questioned^1^. Yet the failure to account for possible species misidentification could affect assessments of population status and distribution and result in erroneous conservation decisions^1^,^18^,^20^.

Few studies have investigated species identification accuracy. In a study of the classification of dinoflagellates, identification accuracy among expert observers was 72%^10^. Thus, more than one in four identifications was, in fact, a *mis*identification. Accuracy also varied dramatically, from 38% to 95%, depending upon the species being identified. However, accuracy was higher and more consistent in expert observers with field expertise than those with expertise gleaned from books^10^. In addition, individual consistency of experts with field expertise averaged 97% accuracy, but for those whose expertise came from books averaged only 75% accuracy. This indicates that observers with field experience were highly consistent in their decision-making (but both for correct and incorrect identifications), whereas the decisions of trained observers without such experience were more variable^10^.

A more recent study focused on the identification of individual mountain bongo antelopes *Tragelaphus eurycerus isaaci* (Thomas 1902) using a matching task^21^. In this task, expert and non-expert observers were shown pairs of pictures of mountain bongos and had to decide whether these depicted the same or different individuals. Under these conditions, experts performed better than non-experts. However, accuracy was far from perfect in both groups of observers, with identification errors in at least one in five trials^21^.

These results suggest that observers can be prone to identification errors in species monitoring. However, whereas one study compared different types of experts during the identification of different species^10^, the other compared experts and non-experts during the identification of individuals from the same species^21^. Consequently, it is still unresolved how experts and non-experts compare directly in species identification.

In this study, we compare the identification accuracy of experts and non-experts with a matching task, in which observers have to decide whether pairs of images depict the same species. A key advantage of this task is that it allows for a direct comparison of observers with expertise in species identification with those without prior knowledge. This approach is used in other research areas, such as the study of forensic human face identification (see ref. 22), as an optimized scenario to establish best-possible performance^23^, but little used in conservation research (although see ref. 21).

In order to investigate the accuracy of species identification in experts and non-experts, the model organisms should also be something that non-experts are familiar with. Bumblebees (*Bombus* sp.) are generally recognisable and attractive to members of the public^24^, and are of great importance to human survival and the economy^25^,^26^,^27^,^28^. Despite this importance, bee populations are in global decline from human activities^26^,^29^,^30^,^31^,^32^. Consequently, bumblebees provide a relevant and timely model for studying the accuracy of species identification in expert and non-expert observers.

Experts and non-experts in bumblebee identification were asked to decide whether 20 pairs of bumblebee images depicted the same or two different species. To increase the relevance of this task to the monitoring of bumblebees by members of the public, the images used in this matching task were coloured illustrations of bumblebees taken from two easily accessible field guides. We sought to explore identification in detail by assessing the overall accuracy of observers in both groups, but also by exploring individual differences and the consistency of identification decisions. For this purpose, participants were asked to classify the same stimuli repeatedly, over three successive blocks.

## Results

### Participant expertise

Experts had substantial knowledge of bumblebees, whereas the non-experts knowledge was minimal. On average, experts named 20.7 bumblebee species (SD = 4.8; min = 15; max = 25), non-expert conservationists (NECs) named on average only 0.4 species (SD = 0.8; min = 0; max = 3), and non-expert non-conservationists (NENCs) only 0.2 species (SD = 0.4; min = 0; max = 1). Similarly, experts correctly chose an average of 19.7/20 UK species from a list of 40 *Bombus* species (Supplementary Table S2) (SD = 0.5; min = 19; max = 20), whereas NECs could only select an average of 1.6 species (SD = 2.1; min = 0; max = 7) and NENCs only 0.1 species (SD = 0.3; min = 0; max = 1).

### Bee matching accuracy

**Figure 1:**
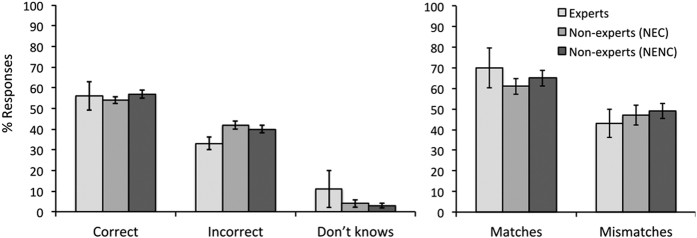
Percentage (±1 s.e.) for correct, incorrect and “don’t know” responses (left graph), and accuracy (±1 s.e.) for match and mismatch pairs (right graph) as a function of expertise. Overall accuracy is low and comparable (54% to 57%) between expert groups.

Overall accuracy in the matching task was low and similar across groups of expertise (Fig. 1), with the mean percentage of correct responses ranging from 54% to 57%. Correspondingly, incorrect responses were high and recorded on between 33% (for experts) and 42% (NEC) of trials across groups. Finally, experts made “don’t know” responses on 11% of trials, while this contributed to less than 5% of responses in both groups of non-experts.

There was no difference between the three participant groups (E, NEC, and NENC) in terms of correct responses (*F*(2,44) = 0.45, *p* = 0.638), incorrect responses (*F*(2,44) = 2.89, *p* = 0.066), and “don’t know” responses (*F*(2,44) = 0.35, *p* = 0.704). Thus, experts and non-experts overall accuracy did not differ on this task. Match performance was similar across expertise groups, at between 61% and 70% accuracy, while mismatch performance was generally lower, at between 43% and 49% accuracy, but similar across the groups (Fig. 1). In line with these observations, a 3 (group: E, NEC, NENC) x 2 (trial type: match, mismatch) mixed-factor ANOVA found an effect of trial type (*F*(1,44) = 13.50, *p* = 0.001), but not of expertise, (*F*(2,44) = 0.61, *p* = 0.545), and no interaction between factors (*F*(2,44) = 0.59, *p* = 0.557).

### Experience and matching accuracy

Percentage accuracy of correct, incorrect and “don’t know” responses were correlated with the years of experience that all participants reported in the identification of UK bumblebees. Correct and incorrect responses declined with experience (*r* = −0.27, *n* = 47, *p* = 0.072 and *r* = −0.30, *n* = 47, *p* = 0.038, respectively), but “don’t know” responses increased with experience (*r* = 0.54, *n* = 47, *p* < 0.001). This suggests that the more experienced observers were less likely to commit to a correct or incorrect identification decision. This inference is drawn tentatively, considering the limited sample of experts and possible extreme scores in the data (see *Participants* section).

### Experience and accuracy for individual items

**Figure 2:**
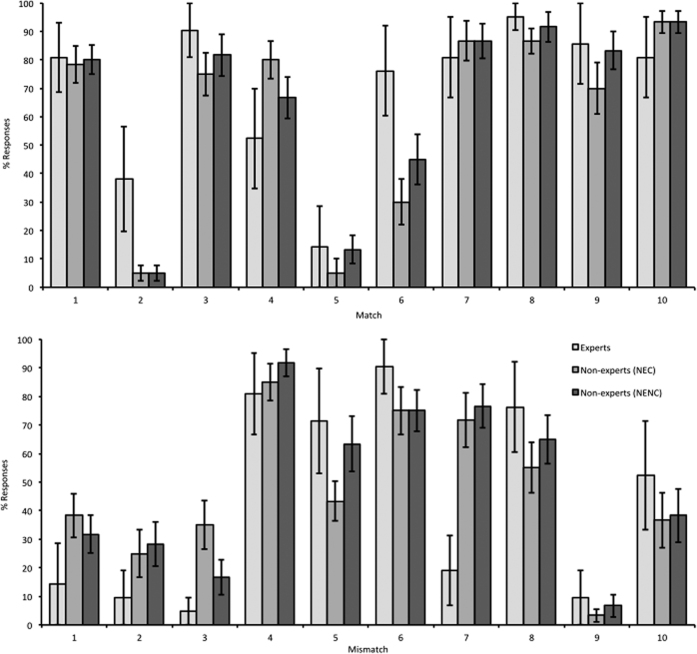
Mean accuracy (±1 s.e.) across groups for each match (top) and mismatch (bottom) image. Effects of expertise were present for only three of these images (match 2, match 6 and mismatch 7).

Accuracy was also calculated for all individual stimulus pairs and the groups of observers. For this by-item analysis, accuracy was combined across the three presentations of each stimulus pair (Fig. 2). One factor ANOVAs for each match and mismatch stimulus show that effects of expertise were present for only three of the 20 images. Post-hoc Tukey tests reveal that experts outperformed non-experts with Match 2 (*F*(2,44) = 5.92, *p* = 0.005; E v NEC and E v NENC *p* = 0.007) and Match 6 (*F*(2,44) = 3.76, E v NEC *p* = 0.024). Conversely, non-experts outperformed experts with Mismatch 7 (*F*(2,44) = 7.00, NEC v E *p* = 0.005 and NENC v E *p* = 0.002). This pattern suggests that the reliance on purely visual information by non-experts generally leads to comparable and occasionally even better accuracy than experts. This indicates that the additional subject-specific experience of experts does not consistently improve, and might even hinder, the matching of some bumblebee species. In some cases, however, expert knowledge also adds a performance advantage that must transcend the available visual information.

### Consistency

We also sought to determine whether experts might be more consistent than non-experts in their identification of bumblebees, by assessing performance across the three repeated trials. Consistent decisions were defined as instances in which observers made the same responses to bumblebee pairs in all three trials.

**Figure 3:**
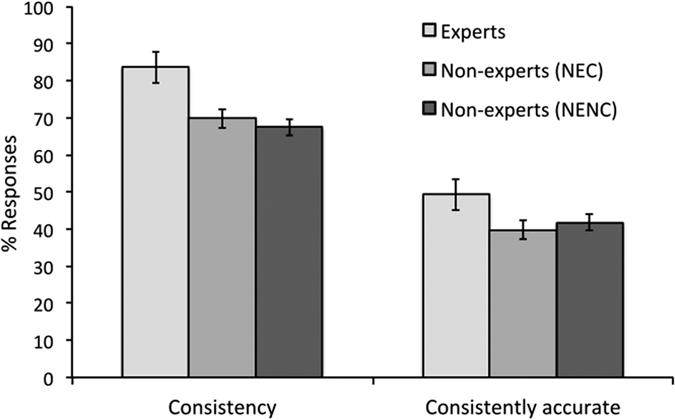
Percentage (±1 s.e.) consistency in responses across presentations of stimuli. Experts were more consistent than non-experts in both overall and accurate answers.

**Figure 4:**
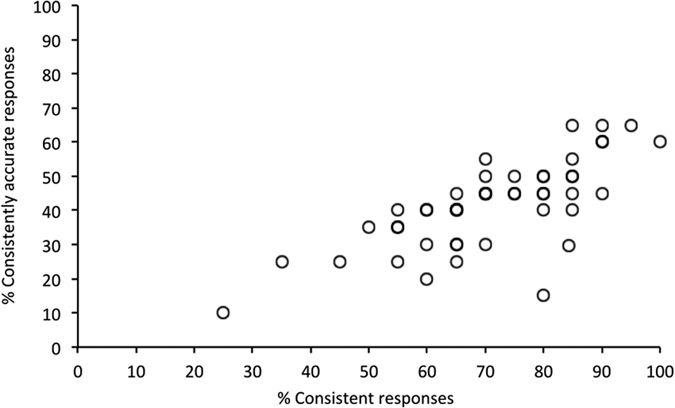
Correlation of consistent and consistently accurate responses. Individuals with consistent responses are more likely to be consistently accurate.

Two consistency measures were obtained. The first of these reflects overall consistency regardless of accuracy, and was calculated by collapsing consistent correct (42% of all decisions), incorrect (27%), and don’t know (2%) responses for the different expertise groups (Fig. 3). A one-factor ANOVA showed that consistency varied between participant groups (*F*(2,44) = 5.42 *p* = 0.008). Tukey post-hoc test showed that experts were more consistent than the NEC (*p* = 0.020) and the NENC groups (*p* = 0.006). The consistency of NECs and NENCs did not differ (*p* = 0.830). A second consistency measure was calculated, which reflects the consistency of accurate responses only. This revealed a similar pattern, with experts outperforming the two non-expert groups. A one-factor ANOVA showed that these differences between groups were not reliable (*F*(2,44) = 0.55, *p* = 0.583). However, a correlation between consistent and consistently-accurate responses (Fig. 4) was found (*r* = 0.722, *n* = 47, *p* < 0.001). Taken together, these data indicate that experts are generally more consistent than non-experts in their responses, but not in their accurate responses. However, the individuals (experts or non-experts) whose responses are more consistent are also more likely to be consistently accurate.

## Discussion

This study examined experts’ and non-experts’ accuracy in the identification of bumblebees. The naming tasks revealed clear effects of expertise, with experts naming an average of 20.7 UK bumblebee species and selecting 19.7/20 from a list of bumblebee species, while NECs and NENCs could name on average less than one species and selected less than two. A different picture emerged when the *actual* visual identification accuracy of these observers was assessed with the matching task. In this task, experts’ overall accuracy was low, at 56%, and indistinguishable from NECs and NENCs. This finding was confirmed when performance was broken down into match and mismatch trials, for which expert and non-expert performance also did not differ. Participants’ self-reported years of experience in bumblebee identification was also correlated with responses on the matching task. This analysis shows that both correct and incorrect responses decline with experience, but ‘don’t know’ responses increase. Thus, observers appear to become more cautious with experience and less willing to commit to any identification decisions. These inferences are drawn tentatively, due to the limited availability of bumblebee experts for this study (*n* = 7). Crucially, however, these findings suggest once again that expertise does not improve the visual identification of bumblebees in the present task.

Overall, these findings converge with studies that have shown that visual species identification can be surprisingly error-prone. In contrast to previous studies, which either examined different types of experts during the identification of different species^10^, or experts and non-experts during the identification of individuals from the same species^21^, the current experiment compared experts and non-experts during species identification. The error rates that are observed across these studies raise important questions concerning the accuracy of species identification using field guides. Such identifications are used for supporting a wide range of actions, such as the monitoring of endangered species^33^,^34^,^35^ and the drafting of appropriate management plans^36^,^37^,^38^. An understanding of error rates needs to be factored into such important conservation activities.

We draw these conclusions with some caveats. It is conceivable, for example, that accuracy among experts and non-experts will vary depending on how images are presented or which guidebooks are at hand. A number of identification guides exist for UK bumblebees, providing a variety of pictures of bumblebees, e.g. line or colour drawings, photographs and stylised diagrams. The extent to which illustrations from different guides accurately capture the key visual features of different bumblebee species and also match each other remains open to exploration^39^, jbut is bound to affect bumblebee identification tasks. Variation in the specimens used by illustrators may also be due to phenotypic variation or even mislabelling in the collections used. Moreover, a difficult question for illustrators is which individual of a species should be drawn in order for a guidebook to represent a ‘typical’ specimen. There is the option to use the holotype (the single specimen on which the species is described), but this may not be readily available or representative of the current UK population. There is the added complication of physical differences caused by age, such as the loss of hair and fading of colour due to the sun^24^.

There is a long history of members of the public contributing to species monitoring programmes^3^,^40^,^41^. An analysis of accuracy for individual items indicated that expert and non-expert accuracy was similar for most bumblebee species here. There were also instances in which experts outperformed non-experts or the reverse pattern was found. This indicates that for some species comparisons, the reliance on purely visual information (as available to both experts and non-experts) produces best accuracy. In other cases, the additional subject-specific expert knowledge can occasionally interfere with the visual identification process. However, expert knowledge can also transcend the available visual information in some cases and provide a benefit in performance. This mixture of results is an intriguing outcome that is perhaps counter to intuition, because it suggests that the identification accuracy of bumblebees might be optimized best by using expert and non-expert decisions in a complementary fashion.

Experts were more consistent in their decisions when the tests were repeated. This effect was only reliable when correct, incorrect and don’t know decisions were combined. Overall, however, the more consistent observers were also more consistently accurate. Thus, experts’ decision criteria appear to be more stable and this might confer an advantage when identification of the same species is assessed repeatedly. Further, systematic investigations of these different effects (visual vs. expertise-driven identifications, consistency) might inform training that is designed to enhance the accuracy of observers. This could help to reduce error rates and improve species monitoring in the field.

Additional identification cues might also be available that could specifically enhance expert performance in practical settings, such as context, behaviour, flight period, or even the presentation of a live or dead specimen. Experts will also have access to additional resources to support identification, such as taxonomic revisions with identification keys and diagrams or natural history collections. We included a short questionnaire in our study to assess field guide usage, which showed that all seven experts reported a combination of up to five field guides, and three also utilised smartphone apps. However, our data suggests that this experience did not enhance performance in the current experiment. More generally, it remains unresolved whether sufficient numbers of experts can be found for research to provide the volumes of data required to understand such factors^42^. Results may also differ for other taxa, but the growth of citizen science and the increase in use of these volunteer data means that species observations, such as those used to inform conservation practitioners, are likely to be heavily reliant on images, either as submissions by non-experts or validation by experts.

In conclusion, this study shows that experts and non-experts both make many errors when using standard field guide illustrations to identify species. This raises important questions surrounding the accuracy of species observations in ecological datasets, and suggests that consideration should be given to possible inaccuracies when such information is used to inform decision makers.

## Methods

### Participants

This research was approved by the Ethics Committee of the School of Psychology at the University of Kent (UKC) and conducted in accordance with the ethical guidelines of the British Psychological Association. Informed consent was obtained from all participants before taking part in the survey. A total of 47 people participated in the survey, comprising expert and non-expert observers. Seven experts (3 female, 4 male, mean age = 40 years, range 25–64) were recruited via a national non-governmental organisation (NGO) specialising in the conservation of bumblebees. Forty non-experts were recruited via the School of Anthropology and Conservation at UKC (30 female, 10 male, mean age = 35 years, range = 18–65). Half of these participants (*n* = 20; 15 female, mean age = 33 years) had a general background in nature conservation and were classified as non-expert conservationists (NEC). The remaining participants (*n* = 20; 15 female, mean age = 37 years) had little or no experience with nature conservation and were therefore classified as non-expert non-conservationists (NENC). All 47 participants reported good vision or corrected-to-normal.

The seven expert participants reported a total of 39 years experience (1–15 years) in the identification of bumblebee species, whereas only seven of the non-experts reported any experience in the identification of bumblebees, ranging from 1 to 8 years. To define this experience further, all participants were asked to evaluate their identification experience on a five-point scale. Self-evaluated bumblebee identification abilities of experts and non-experts did not overlap. Non-experts reported ‘no experience’ (*n* = 33), ‘little experience’ (6) and ‘some experience’ (1), while experts described themselves as ‘experienced’ (3) and ‘competent’ (4).

### Stimuli

The stimuli consisted of 20 pairs of images of bumblebees, comprising 10 match pairs (same species shown), and 10 mismatch pairs (different species shown), using images from two different field guides^43^,^44^. For match pairs, illustrations were from different artists (see Supplementary Table S1). Images in each pair consisted of colour illustrations of dorsal views of entire bumblebees, presented side-by-side on a white background. The paired images always displayed the same caste, e.g. both males, both queens etc. Stimuli were designed to be viewed on a computer monitor, and measured approximately 24 × 15 cm onscreen. No zoom function was included in the survey. Species names were taken from a checklist of extant, native bumblebees recorded in Britain and Northern Ireland (genus *Bombus* Latreille), downloaded from the Natural History Museum (London) website (www.nhm.ac.uk). For each of the species on this list, the BirdGuide application (‘app’) “Bumblebees of Britain and Ireland” (www.birdguides.com) was used to identify phenotypes associated with each species. For species that exhibited a different phenotype according to caste, an individual entry was listed for every caste that differed in appearance from other castes for each species in that guide. Although listed in the guide, *B. pomorum* (Panzer, 1805) and *B. cullumanus* (Kirby, 1802) are believed extinct, and so were removed. The randomised list also included two species in the *lucorum* complex, *B. magnus* (Vogt, 1911) and *B. cryptarum* (Fabricius, 1775), but as research shows that these are visually inseparable^28^, these were also removed. The final list comprised 45 entries representing different UK species and castes where applicable. Twenty entries were randomly sampled from the list for use in the tests. For the survey, the list of the 20 selected entries was randomised again, and the first 10 entries were the species and caste used to create match pairs. The remaining 10 entries on the list formed the first half of a mismatch pair, with the second half of the pair being selected from the other 19 species named on the list. The second species was chosen as so to create a mix of visually similar and dissimilar species (Supplementary Table S1). This set of 10 match and 10 mismatch images was used to create an online survey.

### Procedure

In the experiment, participants’ bumblebee knowledge was initially recorded using two simple tasks to assess their expertise. First, participants were asked to write down all bumblebee species found in the UK. Then participants were asked to select UK bumblebee species from a list of 40 bumblebees (20 UK and 20 non-UK species) (Supplementary Table S2). On completion of the initial assessments, participants were given the matching task. In this task, participants were asked to classify each pair of bumblebees as the same species, two different species, or provide a “don’t know” response using three different buttons on a standard computer keyboard. No time limit was applied to this task to encourage best-possible performance. Participants completed three blocks of this task. Each of these comprised the 10 match and 10 mismatch pairs, and the order of presentation was randomised for the two repeats. In the experiment, each stimulus was therefore shown three times. Inferential statistics were performed using arcsine square-root transformed data.

## Additional Information

**How to cite this article**: Austen, G. E. *et al.* Species identification by experts and non-experts: comparing images from field guides. *Sci. Rep.* **6**, 33634; doi: 10.1038/srep33634 (2016).

## Supplementary Material

Supplementary Information
