# Supplementary material for: Species identification by experts and non-experts: comparing images from field guides
Source: Sci Rep. 2016 Sep 20;6:33634. doi: 10.1038/srep33634 (PMC5028888; doi:10.1038/srep33634)
Supplement: Supplementary Information [file srep33634-s1.pdf]

Species identification by experts and non-experts: comparing images from field guides

Supplementary material

*G. E. Austen[1] (gea7@kent.ac.uk), M. Bindemann[2], R. A. Griffiths[1], D. L. Roberts[1]

[1]Durrell Institute of Conservation and Ecology, School of Anthropology and Conservation, Marlowe Building, University of Kent, Canterbury CT2 7NR, UK

[2] School of Psychology, Keynes College, University of Kent, Canterbury CT2 7NP, UK

Supplementary Table S1. *Bombus* species and caste, and relevant illustrator for each image, used in the survey.

**Image Type**	**Species and caste**	**Species and caste**	**Artist**	
			**Image on left**	**Image on right**
**Match**	*B. distinguendis* (Morawitz 1869) (Queen)		C. Shields	A.J. Hopkins
**Match**	*B. hortorum* (Linnaeus 1761) (Queen)		C. Shields	A.J. Hopkins
**Match**	*B. humilis* (Illiger 1806) (Queen)		A.J. Hopkins	C. Shields
**Match**	*B. jonellus* (Kirby 1802) (Queen)		C. Shields	A.J. Hopkins
**Match**	*B. pratorum* (Linnaeus 1761) (Male)		C. Shields	A.J. Hopkins
**Match**	*B. ruderarius* (Müller 1765) (Male)		C. Shields	A.J. Hopkins
**Match**	*B. ruderarius* (Müller 1765) (Queen)		A.J. Hopkins	C. Shields
**Match**	*B. soroensis* (Fabricius 1777) (Queen)		A.J. Hopkins	C. Shields
**Match**	*B. sylvarum* (Linnaeus 1761) (Queen)		C. Shields	A.J. Hopkins
**Match**	*B. terrestris* (Linnaeus 1758) (Queen)		A.J. Hopkins	C. Shields
**Mismatch**	*B. hortorum* (Linnaeus 1761) (Queen)	*B. ruderatus* (Fabricius 1775) (Queen)	C. Shields	C. Shields
**Mismatch**	*B. lapidarius* (Linnaeus 1758) (Male)	*B. ruderarius* (Müller 1765) (Male)	A.J. Hopkins	A.J. Hopkins
**Mismatch**	*B. lapidarius* (Linnaeus 1758) (Queen)	*B. ruderarius* (Müller 1765) (Queen)	C. Shields	C. Shields
**Mismatch**	*B. lucorum* (Linnaeus 1761) (Queen)	*B. terrestris* (Linnaeus 1758) (Queen)	A.J. Hopkins	C. Shields
**Mismatch**	*B. lucorum* (Linnaeus 1761) (Queen)	*B. soroensis* (Fabricius 1777) (Queen)	C. Shields	C. Shields
**Mismatch**	*B. monticola* (Smith 1849) (Queen)	*B. lapidarius* (Linnaeus 1758) (Queen)	A.J. Hopkins	A.J. Hopkins
**Mismatch**	*B. muscorum* (Linnaeus 1758) (Male)	*B. humilis* (Illiger 1806) (Male)	C. Shields	C. Shields
**Mismatch**	*B. pascuorum* (Scopoli 1763) (Male)	*B. muscorum* (Linnaeus 1758) (Male)	C. Shields	C. Shields
**Mismatch**	*B. pascuorum* (Scopoli 1763) (Queen)	*B. sylvarum* (Linnaeus 1761) (Queen)	A.J. Hopkins	C. Shields
**Mismatch**	*B. subterraneus* (Linnaeus 1758) (Queen)	*B. monticola* (Smith 1849) (Queen)	A.J. Hopkins	C. Shields

Supplementary Table S2. List of UK and Palaearctic species of the genus *Bombus*.

argillaceus	*hypnorum*	*ruderarius*
armeniacus	*hypocrita*	*ruderatus*
atripes	*jonellus*	*rupestris*
barbutellus	*ladakhensis*	*schrencki*
bohemicus	*lapidarius*	*soroeensis*
campestris	*lucorum*	*subterraneus*
cryptarum	*margreiteri*	*sylvarum*
cullumanus	*monticola*	*sylvestris*
deuteronymus	*morawitzianus*	*terrestris*
fragrans	*muscorum*	*tichenkoi*
hedini	*opulentus*	*ussurensis*
hortorum	*pascuorum*	*wurflenii*
humilis	*perezi*	
hyperboreus	*persicus*	
